# MAPKAPK5-AS1 drives the progression of hepatocellular carcinoma via regulating miR-429/ZEB1 axis

**DOI:** 10.1186/s12860-022-00420-x

**Published:** 2022-04-25

**Authors:** Zongqing Peng, Xinhua Ouyang, Yexing Wang, Qiming Fan

**Affiliations:** Department of General Surgery, Xiangyang Central Hospital, Affiliated Hospital of Hubei University of Arts and Science, Zhongyuan Road No.7, Xiangyang, 441000 Hubei Province China

**Keywords:** Hepatocellular carcinoma, MAPKAPK5-AS1, miR-429, ZEB1

## Abstract

**Background:**

Hepatocellular carcinoma (HCC) is a common malignancy. Long non-coding RNAs (lncRNAs) partake in the progression of HCC. However, the role of lncRNA MAPKAPK5-AS1 in the development of HCC has not been fully clarified.

**Methods:**

RNA sequencing data and quantitative real-time polymerase chain reaction (qRT-PCR) were adopted to analyze MAPKAPK5-AS1, miR-429 and ZEB1 mRNA expressions in HCC tissues and cell lines. Western blot was used to detect ZEB1, E-cadherin and N-cadherin protein expressions. 3-(4,5-dimethylthiazol-2-yl)-2,5-diphenyltetrazolium bromide (MTT), Transwell and flow cytometry assays were adopted to analyze the effects of MAPKAPK5-AS1 on cell proliferation, migration, invasion and apoptosis. Besides, luciferase reporter assay was used to detect the targeting relationship between miR-429 and MAPKAPK5-AS1 or ZEB1 3’UTR. The xenograft tumor mouse models were used to explore the effect of MAPKAPK5-AS1 on lung metastasis of HCC cells.

**Results:**

MAPKAPK5-AS1 and ZEB1 expressions were up-regulated in HCC tissues, and miR-429 expression is down-regulated in HCC tissues. MAPKAPK5-AS1 knockdown could significantly impede HCC cell proliferation, migration, invasion and epithelial-mesenchymal transition (EMT), as well as promote cell apoptosis. MAPKAPK5-AS1 overexpression could enhance L02 cell proliferation, migration, invasion and EMT, and inhibit cell apoptosis. MiR-429 was validated to be the target of MAPKAPK5-AS1, and miR-429 inhibitors could partially offset the effects of knocking down MAPKAPK5-AS1 on HCC cells. MAPKAPK5-AS1 could positively regulate ZEB1 expression through repressing miR-429. Moreover, fewer lung metastatic nodules were observed in the lung tissues of nude mice when the MAPKAPK5-AS1 was knocked down in HCC cells.

**Conclusion:**

MAPKAPK5-AS1 can adsorb miR-429 to promote ZEB1 expression to participate in the development of HCC.

**Supplementary Information:**

The online version contains supplementary material available at 10.1186/s12860-022-00420-x.

## Introduction

Primary liver cancer is the third leading cause of cancer-related deaths. As the most common pathological type of liver cancer, hepatocellular carcinoma (HCC) is an aggressive malignancy with a poor prognosis [1]. Although significant advances have been made in treatment methods such as surgery, liver transplantation, chemotherapy, target therapy and immunotherapy, the 5-year survival rate of HCC patients after radical resection is only 10–15% [2, 3]. Therefore, the molecular mechanism of HCC progression needs to be accurately identified.

Long non-coding RNAs (lncRNAs) are non-coding RNA transcripts, with a length of over 200 nucleotides, that do not have the ability to encode proteins [4]. In recent years, accumulating studies confirm that lncRNA is involved in the progression of many malignancies including HCC [5–9]. For example, lncRNA MIAT can promote HCC cells proliferation and invasion by sponging miR-214 [7]. LncRNA-PDPK2P enhances HCC cell proliferation, migration and invasion through the PDK1/AKT/Caspase 3 pathway [8]. LncRNA MCM3AP-AS1 knockdown suppresses HCC cell proliferation, colony formation and cell cycle progression, and induces apoptosis [9]. Therefore, lncRNAs are considered as potential targets for HCC treatment. MAPKAPK5-AS1 is abnormally expressed in colorectal cancer tissues and is related to the progression of colorectal cancer [10]. By analyzing the TCGA samples, we found that MAPKAPK5 antisense RNA 1 (MAPKAPK5-AS1) expression was upregulated in HCC tissues and related with the advanced stages and poor overall survival of HCC patients. Nevertheless, the role of MAPKAPK5-AS1 in the development of HCC is far from been fully clarified.

MicroRNAs (miRNAs) are single-stranded RNAs with a length of 18–22 nucleotides. They can modulate expressions of various genes by binding to the 3′-untranslated region (3’UTR) of target mRNAs [11]. Many miRNAs participate in regulating cell differentiation, proliferation, apoptosis and other biological processes of HCC cells, such as miR-490-3p, miR-29a, and miR-449a [12–14]. Among them, miR-429 expression is significantly down-regulated in HCC tumor tissues, and miR-429 can inhibit HCC cell growth, migration and invasion, and promote apoptosis [15, 16]. We searched the Starbase database and found that miR-429 contained the complementary binding site with MAPKAPK5-AS1. Besides, it was revealed that miR-429 expression was downregulated in HCC tissues compared with the normal tissues in TCGA. These data implied that MAPKAPK5-AS1 could probably interact with miR-429 to modulate its expression in HCC.

Zinc finger E-box binding homeobox 1 (ZEB1) (also known as TCF8) is a member of ZEB family. The ZEB family is involved in embryonic development, for example, inducing epithelial-mesenchymal transition (EMT) of the cells [17]. During EMT, epithelial cells lose polarity and become invasive, and this process is considered as a pivotal biological event during the metastasis of cancer cells [18]. ZEB1 is a vital regulator in HCC progression [19]. Reportedly, SIAH1 can sponge miR-3129-5p and upregulate ZEB1 expression, thus mediating the doxorubicin resistance of HCC cells [20]. In this study, UALCAN database and GEPIA database showed the ZEB1 was highly expressed in HCC tissues compared with normal tissues, which was correlated with the poor overall survival of HCC patients.

LncRNAs can function as miRNA sponges [competing endogenous RNAs (ceRNAs)] to regulate gene expression [21]. In this study, MAPKAPK5-AS1 expression in HCC tissues and cell lines was detected, and the interactions of MAPKAPK5-AS1, miR-429 and ZEB1 in HCC development were explored. We hypothesized that MAPKAPK5-AS1 could regulate HCC progression via modulating miR-429 and ZEB1, and this study was performed to verify this scientific hypothesis.

## Materials and methods

### Samples collection

From November 2018 to June 2019, 36 cases of tumor tissues and matched adjacent tissues of HCC patients diagnosed by histopathological biopsy were collected from Xiangyang Central Hospital, and the samples were frozen in liquid nitrogen and then stored at − 80 °C. Patients with a history of preoperative anti-cancer treatments, such as radiotherapy or chemotherapy, immunotherapy and traditional Chinese medicine, were excluded from the research. All patients signed informed consent, and this study complied with the Helsinki Declaration and was backed by the Ethics Committee of Xiangyang Central Hospital.

### Cell culture and transfection

Human immortalized liver cell line L02, human HCC cell lines HepG2 and Huh7, and kidney epithelial cell 293 T cells were obtained from BioVector NTCC Inc. (Beijing, China). These cells were cultivated in Dulbecco’s Modified Eagle’s Medium (DMEM, Gibco, Grand Island, NY, USA) with 10% fetal bovine serum (FBS, Gibco, Grand Island, NY, USA), 100 units/ml penicillin and 100 μg/ml streptomycin at 37 °C in 5% CO_2_. Two short hairpin RNA (shRNA) targeting MAPKAPK5-AS1 (sh-MAPKAPK5-AS1#1 and sh-MAPKAPK5-AS1#2) and shRNA scramble control (sh-NC) were constructed by Genepharma (Shanghai, China); miR-429 mimics, miR-429 inhibitors and its corresponding negative control (miR-NC) were obtained from RiboBio (Guangzhou, China). They were transfected into the cells using Lipofectamine 2000 reagent (Invitrogen, Carlsbad, CA, USA) in line with the instructions and collected after 24 h for subsequent experiments.

### RNA extraction and quantitative real-time polymerase chain reaction (qRT-PCR)

TRIZol reagent (Invitrogen, Carlsbad, CA, USA) was adopted to extract RNA from HCC tissues, adjacent tissues and cells, and the M-MLV Reverse Transcriptase kit (Invitrogen, Carlsbad, CA, USA) was exerted to synthesize complementary DNA (cDNA). Besides, a SYBR Premix Ex Taq kit (Takara, Dalian, China) was used for qRT-PCR. Relative expressions of the genes were determined by 2^−ΔΔCT^ method, and glyceraldehyde-3-phosphate dehydrogenase (GAPDH) and U6 were adopted as internal references. The primer sequences: MAPKAPK5-AS1: forward 5′-AAGCCCGAGTCTGATGCTAA-3′, and reverse 5′-CTGCACACCTCTTCTGGTCA-3′; miR-429: forward 5′-UAAUACUGUCUGGUAAAACCGU-3′, and reverse 5′-UCUCCGAACGUGUCACGUTT-3′; ZEB1: forward 5′- GATGATGAATGCGAGTCAGATGC-3′, and reverse 5′-ACAGCAGTGTCTTGTTGTTGT-3′; U6 forward 5′-CTCGCTTCGGCAGCACA-3′, and reverse 5′-ACGCTTCACGAATTTGCGT-3′; GAPDH forward 5′-AACTTTGGCATTGTGGAAGG-3′, and reverse, 5′-ACACATTGGGGGTAGGAACA-3′.

### Western blot

Total proteins of HCC tissues and cells were extracted by RIPA buffer (Beyotime, Shanghai, China) in ice, and quantified by a BCA kit (Millipore, Billerica, MA, USA). Then the protein samples were mixed with loading buffer and heated in boiling water. The same amount of protein was separated by SDS-PAGE, transferred to PVDF membrane (Millipore, Billerica, MA, USA) and blocked with 5% skim milk at room temperature for 2 h. The primary antibodies of ZEB1 (1: 1200, ab203829, Abcam, Cambridge, UK), E-cadherin (1: 1500, ab133597, Abcam, Cambridge, UK), N-cadherin (1: 1500, ab207608, Abcam, Cambridge, UK) and GAPDH (1: 1500, ab37168, Abcam, Cambridge, UK) were used to incubate with membrane at 4 °C for 8 h. HRP-goat anti-rabbit (IgG) secondary antibody (1:1400; ab6721; Abcam, Cambridge, UK) was then incubated with the membrane at 24 °C for 2 h, and protein signals were visualized using ECL luminescence reagent (Sangon, Shanghai, China).

### Cell proliferation assay

Transfected cells were transferred into a 96-well plate at 37 °C and 5% CO_2_ overnight and then incubated with 10 μL of 5 mg/mL 3-(4,5-dimethylthiazol-2-yl)-2,5-diphenyltetrazolium bromide (MTT) reagent (Sigma, MO, USA) at 37 °C for 4 h. After being centrifugated, the supernatant was discarded, the samples were mixed in 150 μL of DMSO (Sigma, MO, USA), and the plate was shaken gently to dissolve the formazan. Eventually, an MRX II absorbance reader (DYNEX Technologies, Chantilly, VA, USA) was used to measure the absorbance at 490 nm wavelength.

### Cell migration and invasion assay

Transwell chambers (Corning, Corning, NY, USA) were employed to observe cell migration and invasion in accordance with the manufacturer’s instructions. In the migration experiment, the cells, suspended in serum-free medium, were inoculated into the upper chamber, and 500 μL of DMEM with 10% FBS was loaded in the lower chamber. After 24 h, migrating cells were fixed with 4% paraformaldehyde and then stained with 0.2% crystal violet solution for 15 min. The number of cells in a randomly selected field of view was counted under a microscope. In the invasion experiment, Matrigel (BD Bioscience, San Jose, CA, USA) was pre-coated on the filter, and the other procedures were the same with those in the migration assay.

### Flow cytometry

After trypsinization, the transfected HCC cells were re-suspended in a binding buffer. Then, the cells were incubated with 5 μL of Annexin V-FITC staining solution (Annexin V-FITC Apoptosis Detection kit, BD Biosciences, San Jose, CA, USA) and 5 μL of propidium iodide (PI; Sigma-Aldrich, St. Louis, MO, USA) for 15 min in the dark. After the cells were washed by phosphate buffer saline, a flow cytometer (FACSCalibur, BD Biosciences Co., Franklin Lakes, NJ, USA) was used to detect cell apoptosis. The sum of the data of Q2 and Q3 was the apoptosis rate (%).

### Dual-luciferase reporter gene assay

Wild or mutant MAPKAPK5-AS1/ZEB1 3’UTR sequences containing miR-429 binding sequence were synthesized and cloned into pmirGLO Vector (Promega, Shanghai, China). The constructed vector was co-transfected with miR-429 mimics or miR-NC into 293 T cells. After 48 h, luciferase activity was detected with a Dual-Luciferase Reporter Assay System (Promega, Shanghai, China).

### UALCAN database analysis

The UALCAN (http://ualcan.path.uab.edu) is an online web tool for analyzing cancer omics data based on the TCGA data. It was searched for analyzing the relative expressions of MAPKAPK5-AS1, miR-429 and ZEB1 in HCC tissues based on clinical stages or other clinical characteristics. *P* < 0.05 was considered to be statistically significant.

### GEPIA database

GEPIA (http://gepia.cancer-pku.cn/) is an online web tool for analyzing the RNA sequencing expression data of 9736 tumor samples and 8587 normal tissue samples from TCGA. GEPIA database was used to examine the relationship between the expression of MAPKAPK5-AS1 or ZEB1 and the overall survival of HCC patients.

### In vivo experiment

Four-week-old male BALB/c nude mice were randomly divided into two groups (5 mice per group). Then, 5 × 10^6^ sh-MAPKAPK5-AS1#1 or sh-NC was transfected into HepG2 cells, and transfected cells were injected into the tail vein of mice in each group. After 4 weeks, all nude mice were euthanized, and the lungs were surgically removed and fixed in 10% neutral phosphate-buffered formalin, followed by Hematoxylin & Eosin (H&E) staining, and the number of metastases nodules was counted under a microscope.

### Statistical analysis

All experiments were conducted three times, and the data were expressed as mean ± standard deviation (SD). The comparison between different groups was performed by Student’s *t*-test or one-way ANOVA with Tukey post hoc test. The association of MAPKAPK5-AS1 expression with the clinicopathological parameters of HCC patients was measured by chi-square test, and the correlation was measured by Spearman correlation analysis. Statistical analysis was performed using GraphPad Prism 7 software (GraphPad Inc., San Diego, CA, USA). *P* < 0.05 was considered statistically meaningful.

## Results

### MAPKAPK5-AS1 and ZEB1 expressions were elevated in HCC tissues and cell lines, and miR-429 expression was down-regulated

**Fig. 1:**
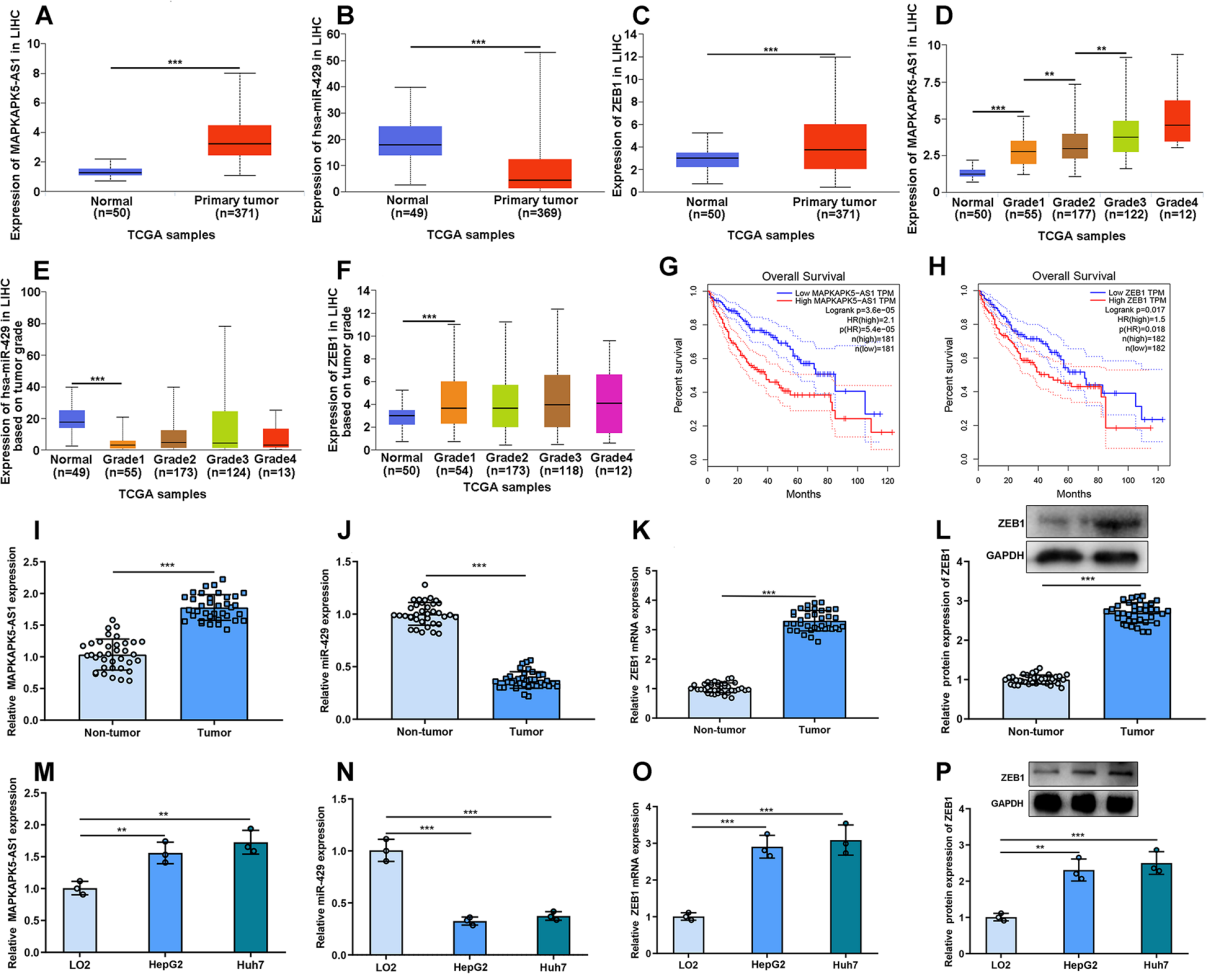
MAPKAPK5-AS1 and ZEB1 expressions were up-regulated in HCC, and miR-429 expression was down-regulated. **A-C** UALCAN database showed the expressions of MAPKAPK5-AS1, miR-429 and ZEB1 in HCC tissues and normal tissues. **D-F** UALCAN database showed the expressions of MAPKAPK5-AS1, miR-429 and ZEB1 in HCC tissues and normal tissues based on different tumor grades. **G-H** GEPIA database showed the relationship between the expression of MAPKAPK5-AS1/ ZEB1 and the overall survival of HCC patients. **I-K** qRT-PCR was used to detect the expressions of MAPKAPK5-AS1, miR-429 and ZEB1 mRNA in HCC tissues and adjacent tissues. **L** Western blot was used to detect the expression of ZEB1 in HCC tissues and adjacent tissues. **M-O** qRT-PCR was used to detect the expressions of MAPKAPK5-AS1, miR-429 and ZEB1 mRNA in HCC cell lines (HepG2 and Huh7) and normal human liver cell lines (L02). **P** Western blot was used to detect the expression of ZEB1 in HCC cell lines (HepG2 and Huh7) and immortalized liver cell line (L02). **P < 0.01; and ***P < 0.001

By searching UALCAN database, we examined the expressions of MAPKAPK5-AS1, miR-429 and ZEB1 in HCC tumor tissues and adjacent normal tissues. The results showed that MAPKAPK5-AS1 and ZEB1 expressions were significantly upregulated in tumor samples, and miR-429 expression was downregulated (Fig. 1A-C). We next analyzed MAPKAPK5-AS1, miR-429 and ZEB1 expressions based on tumor grade. MAPKAPK5-AS1 expression was higher in grade 3 than that in grade 2, and in grade 2 than that in grade 1, as well as in grade 1 that in normal tissues (Fig. 1D). The expression of miR-429 was significantly lower and the expression of ZEB1 was higher in grade 1 tumor tissues than those in normal tissues (Fig. 1E-F). Additionally, it was found that the high MAPKAPK5-AS1 expression or ZEB1 expression was associated with the poor overall survival of HCC patients (Fig. 1G-H).

Subsequently, qRT-PCR or Western blot was adopted to analyze the expressions of MAPKAPK5-AS1, miR-429 and ZEB1 in collected HCC tissues and adjacent tissues, and we found that MAPKAPK5-AS1 and ZEB1 mRNA expressions were considerably elevated in HCC tissues, and miR-429 expression was markedly reduced in HCC tissues (Fig. 1I-L). In addition, we found that the expressions of MAPKAPK5-AS1 and ZEB1 mRNA in HCC-derived cell lines HepG2 and Huh7 were significantly up-regulated compared to L02, and miR-429 expression was significantly lower in HCC-derived cell lines (Fig. 1M-O). Western blot showed that ZEB1 expression was significantly enhanced in HCC-derived cell lines (Fig. 1P). Based on the median expression of MAPKAPK5-AS1 in HCC tissues, 36 patients with HCC were divided into high expression group (*n* = 18) and low expression group (n = 18). Chi-square test showed that high expression of MAPKAPK5-AS1 was associated with larger tumor size, advanced stage and lymph node metastasis (Table 1).

**Table 1:** The relationship between MAPKAPK5-AS1 expression and clinicopathological parameters of HCC

Parameter	Case	MAPKAPK5-AS1 expression		*P* value
		Low (*n* = 18)	High (*n* = 18)	
Age (years)				
≤ 60	20	12	8	0.180
>60	16	6	10	
Gender				
Female	19	10	9	0.738
Male	17	8	9	
Tumor size				
≤ 5 cm	12	9	3	0.034*
>5 cm	24	9	15	
Clinical stages				
I-II	20	13	7	0.044*
III	16	5	11	
Lymphatic metastasis				
Yes	26	10	16	0.026*
No	10	8	2	

### MAPKAPK5-AS1 silencing inhibited HCC cell proliferation, metastasis and EMT

**Fig. 2:**
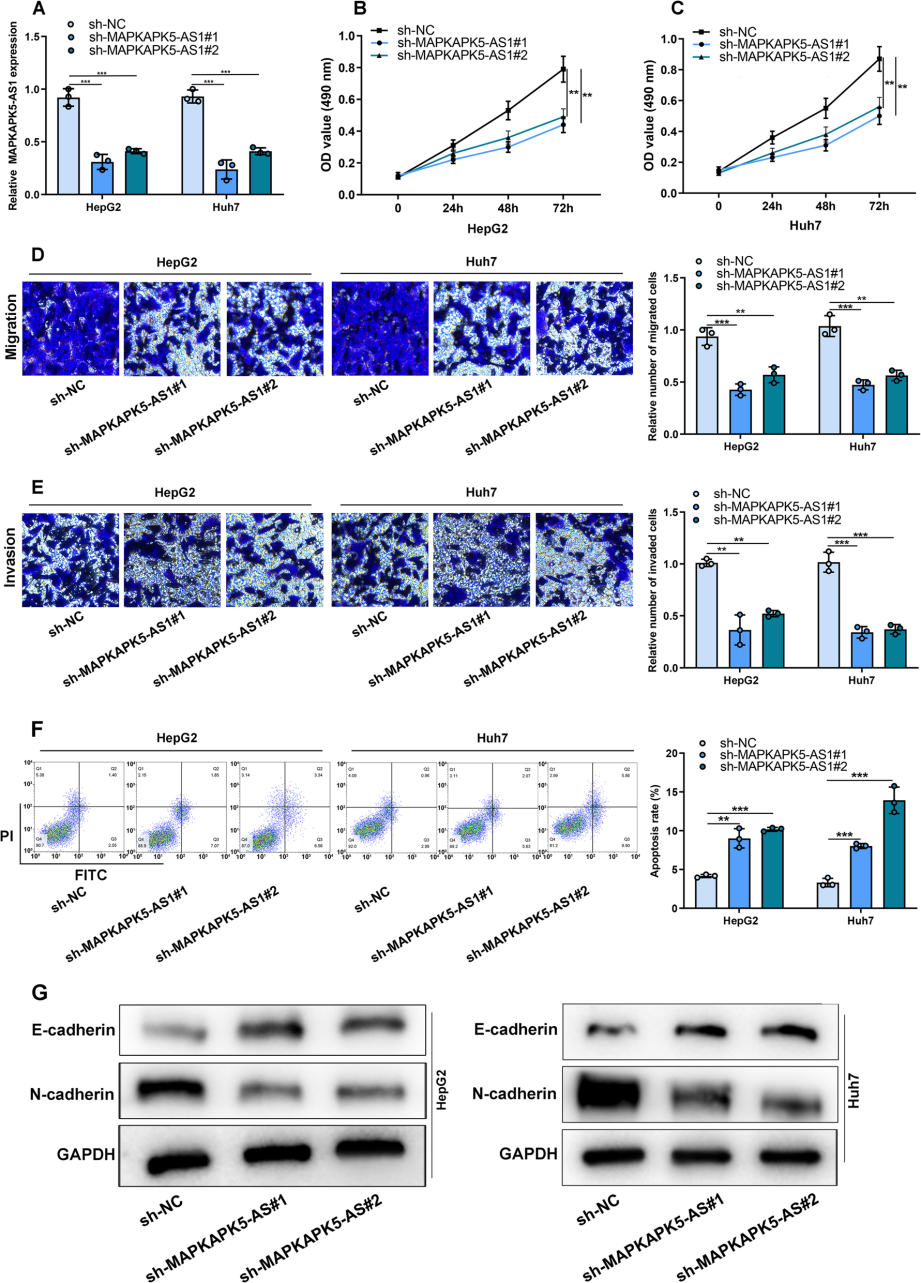
MAPKAPK5-AS1 silencing inhibited HCC cell proliferation, migration and invasion of HCC cells. **A** qRT-PCR was used to detect the expression of MAPKAPK5-AS1 in HepG2 and Huh7 cells. **B-C** MTT assay was adopted to measure the proliferation of HepG2 and Huh7 cells after MAPKAPK5-AS1 was knocked down. **D-E** Transwell assay was adopted to measure the migration and invasion of HepG2 and Huh7 cells after MAPKAPK5-AS1 was knocked down. **F** Flow cytometry was adopted to measure the apoptosis of HepG2 and Huh7 cells after MAPKAPK5-AS1 was knocked down. **G** Western blot was used to detect E-cadherin and N-cadherin expressions in HepG2 and Huh7 cells after MAPKAPK5-AS1 was knocked down. *P < 0.05; **P < 0.01; ***P < 0.001

Two MAPKAPK5-AS1 shRNAs (sh-MAPKAPK5-AS1#1 and sh-MAPKAPK5-AS1#2) were utilized to silence MAPKAPK5-AS1 in HCC cell lines to successfully construct the low expression model of MAPKAPK5-AS1 (Fig. 2A). MTT experiments implied that compared with sh-NC group, knockdown of MAPKAPK5-AS1 markedly inhibited cell proliferation (Fig. 2B-C). Transwell experiments confirmed that depletion of MAPKAPK5-AS1 could significantly inhibit cell migration and invasion (Fig. 2D-E). Flow cytometry showed that suppressing MAPKAPK5-AS1 expression markedly promoted the apoptosis rate of HCC cells (Fig. 2F). Furthermore, knockdown of MAPKAPK5-AS1 significantly suppressed the protein expression level of N-cadherin and increased E-cadherin expression in HepG2 and Huh 7 cells (Fig. 2G). Thus, it could be concluded that suppressing MAPKAPK5-AS1 expression inhibited the proliferation, migration, invasion and EMT of HCC cells.

### Overexpression of MAPKAPK5-AS1 promoted L02 cells proliferation, migration and invasion

**Fig. 3:**
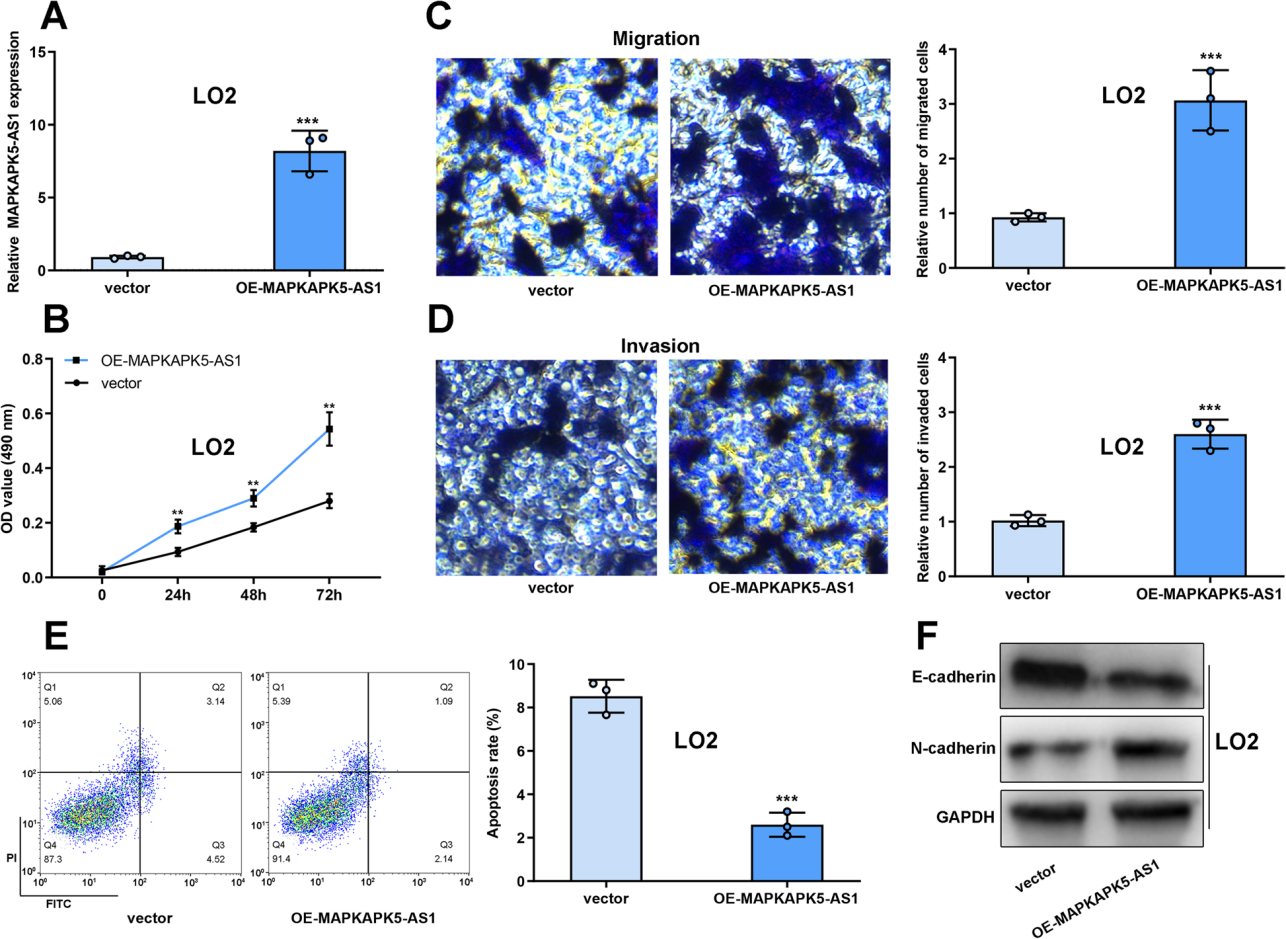
Overexpression of MAPKAPK5-AS1 promoted L02 cells proliferation, metastasis and EMT. **A** qRT-PCR was used to detect the expression of MAPKAPK5-AS1 in L02 cells after MAPKAPK5-AS1 overexpression plasmid was transfected into L02 cells. **B** MTT assay was adopted to measure the proliferation of L02 cells. **C-D** Transwell assay was adopted to measure the migration and invasion of L02 cells. **E** Flow cytometry was adopted to measure the apoptosis of L02 cells. **F** Western blot was used to detect E-cadherin expression and N-cadherin expression in L02 cells. **P < 0.01; ***P < 0.001

To examine the oncogenic properties of MAPKAPK5-AS1, MAPKAPK5-AS1 overexpression plasmid was transfected into L02 cells (Fig. 3A). MTT and Transwell assays showed that overexpression of MAPKAPK5-AS1 could markedly improve the proliferation, migration and invasion of L02 cells compared with empty vector group (Fig. 3B-D). Flow cytometry showed that overexpression of MAPKAPK5-AS1 inhibited the apoptosis of L02 cells (Fig. 3E). Western blot revealed that after overexpressing MAPKAPK5-AS1 in L02 cells, the protein level of N-cadherin was increased and the protein level of E-cadherin was decreased (Fig. 3F). These results suggested that MAPKAPK5-AS1 overexpression promoted malignancy of L02 cells.

### MiR-429 was the target of MAPKAPK5-AS1

**Fig. 4:**
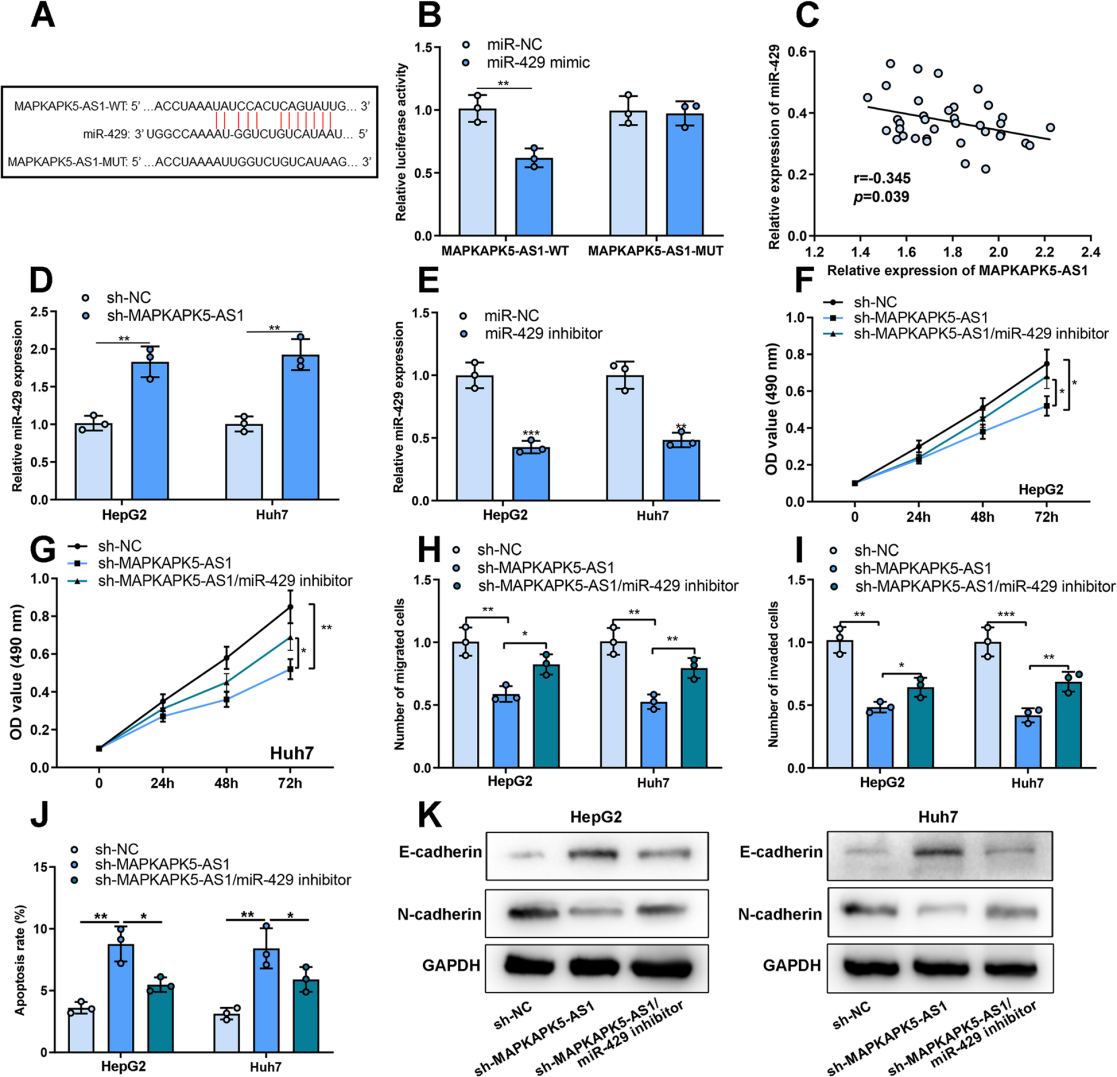
MAPKAPK5-AS1 played a role in HCC by regulating miR-429 expression. **A** The binding site of MAPKAPK5-AS1 and miR-429. **B** Luciferase activity was examined in 293 T cells transfected with MAPKAPK5-AS1-WT or MAPKAPK5-AS1-MUT and miR-3429 mimic or miR-NC. **C** Spearman correlation analysis of MAPKAPK5-AS1 expression and miR-429 expression in HCC tissues. **D** qRT-PCR was used to detect the expression of miR-429 in HCC cells after the cells were transfected with si-MAPKAPK5-AS1 or si-NC. **E** qRT-PCR was used to detect the expression of miR-429 after HCC cells were transfected with miR-429 inhibitors or miR-NC. **F-I** MTT and Transwell assays were adopted to detect the HCC cell proliferation, migration and invasion. **J** Flow cytometry was adopted to detect the HCC cell apoptosis. **K** Western blot was used to measure E-cadherin and N-cadherin expressions in HepG2 and Huh7 cells. *P < 0.05; **P < 0.01; ***P < 0.001

StarBase database suggested that miR-429 was a potential target for MAPKAPK5-AS1 (Fig. 4A). Luciferase reporter assay was performed to prove the direct binding between MAPKAPK5-AS1 and miR-429. Then, we constructed luciferase reporters containing MAPKAPK5-AS1 wild-type binding sites (MAPKAPK5-AS1-WT) or MAPKAPK5-AS1-mutant binding sites (MAPKAPK5-AS1-MUT). The luciferase activity of MAPKAPK5-AS1-WT was significantly inhibited by miR-429. However, the luciferase activity of MAPKAPK5-AS1-MUT was not affected by miR-429 (Fig. 4B). In addition, a negative correlation between miR-429 and MAPKAPK5-AS1 expressions in HCC tissues was observed (Fig. 4C). As against the control group, miR-429 expression in the MAPKAPK5-AS1 knockdown group was significantly increased (Fig. 4D). To confirm that MAPKAPK5-AS1 participated in HCC development by regulating miR-429 expression, we successfully constructed miR-429 low expression model in HCC cell line using miR-429 inhibitors (Fig. 4E). Subsequently, it was revealed that inhibition of miR-429 could significantly reverse the inhibitory effects of MAPKAPK5-AS1 knockdown on the proliferation, migration, invasion, apoptosis and EMT process of HCC cells (Fig. 4F-K).

### MAPKAPK5-AS1 regulated ZEB1 expression by adsorbing miR-429

**Fig. 5:**
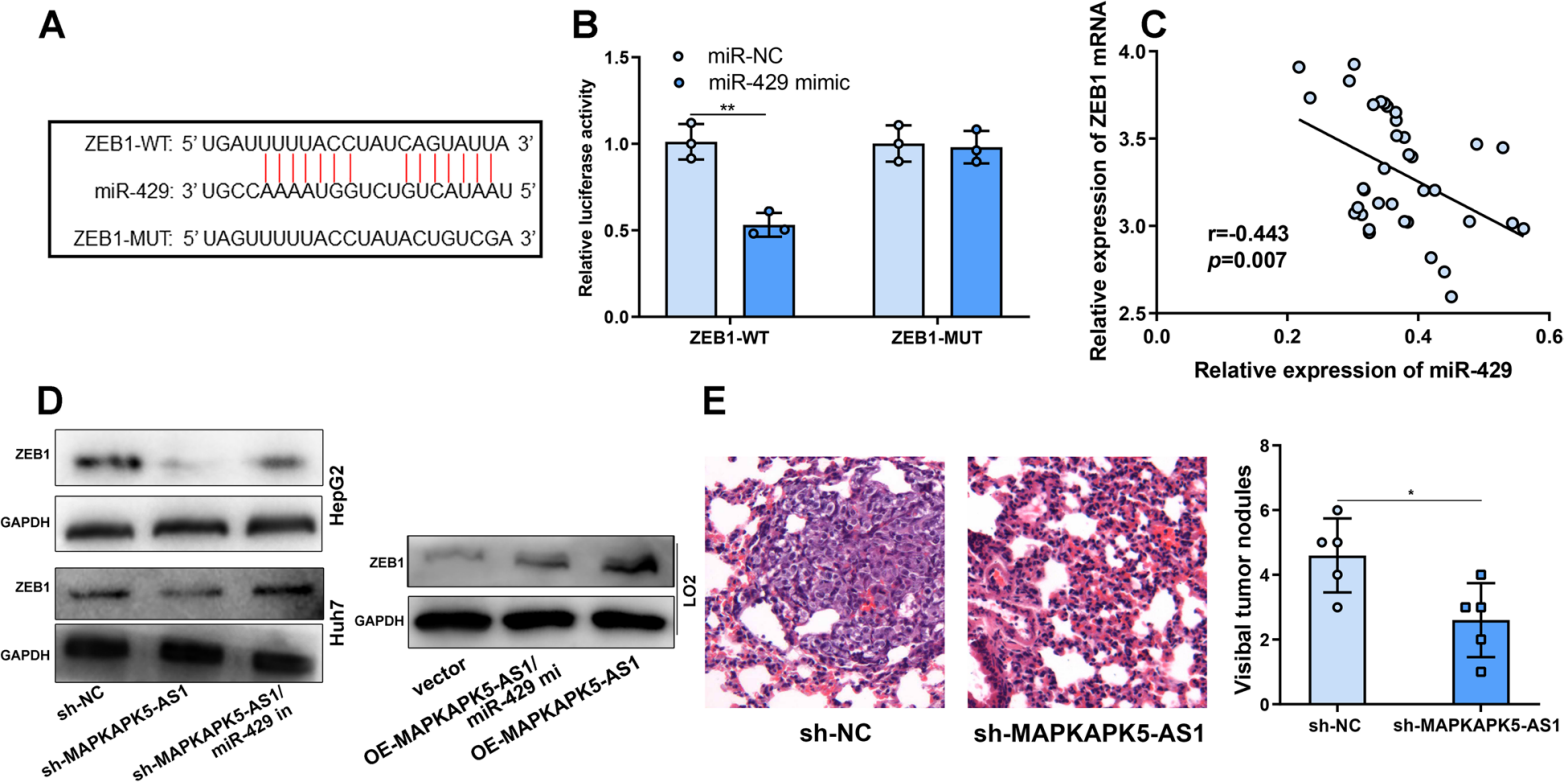
MAPKAPK5-AS1 regulated ZEB1 expression through adsorbing miR-429. **A** The binding site between ZEB1 3’UTR and miR-429 was predicted via bioinformatics analysis. **B** The luciferase reporter assay showed that miR-429 could directly bind to ZEB1 3’UTR. **C** Spearman correlation analysis of ZEB1 mRNA expression and miR-429 expression in HCC tissues. **D** Western blot was used to detect the expression of ZEB1 in HepG2 and Huh7 cells after the transfection of si-MAPKAPK5-AS1 and si-MAPKAPK5-AS1/miR-429 inhibitors and in L02 cells transfected MAPKAPK5-AS1 overexpression plasmid and MAPKAPK5-AS1 overexpression plasmid/miR-429 mimics. **E** Lung metastasis assay in vivo was performed and the number of tumor nodules was counted. *P < 0.05; **P < 0.01

We searched the StarBase database and found that ZEB1 3’UTR contained a binding sequence to miR-429 (Fig. 5A). We further validated the binding relationship between miR-429 and ZEB1 by a luciferase activity reporter experiment. The result showed that overexpression of miR-429 suppressed the luciferase activity of ZEB1-WT reporter but had no effect on the luciferase activity of ZEB1-MUT reporter (Fig. 5B). qRT-PCR showed that there was a negative correlation between the expressions of miR-429 and ZEB1 mRNA in HCC tissues (Fig. 5C). Western blot showed that the expression of ZEB1 was significantly down-regulated after the depletion of MAPKAPK5-AS1 as against the control group, whereas the transfection of miR-429 inhibitors abolished the effects in HCC cells and miR-429 mimic could reverse the effects of MAPKAPK5-AS1 overexpression on ZEB1 expression in L02 cells (Fig. 5D).

### MAPKAPK5-AS1 promoted lung metastasis of HCC cells in vivo

To further determine the tumorigenic potential of MAPKAPK5-AS1 in vivo, HepG2 cells transfected with sh-NC or sh-MAPKAPK5-AS1 were injected into the caudal vein of the nude mice. H&E staining of lung sections showed that knocking down MAPKAPK5-AS1 expression reduced the number of lung metastatic nodules compared with that in the sh-NC group (Fig. 5E). This finding further supported that MAPKAPK5-AS1 modulated the progression of HCC.

## Discussion

It is well documented that lncRNAs play crucial roles in the tumorigenesis and cancer progression, and may be promising biomarkers and therapeutic targets [22]. LncRNA MAPKAPK5-AS1 is abnormally expressed in many tumors, such as colon cancer, glioma and thyroid cancer [10, 23, 24]. In HCC, MAPKAPK5-AS1 upregulated PLAG1 like zinc finger 2 (PLAGL2) expression by acting as a ceRNA to decoy miR-154-5p, thereby activating EGFR/AKT signaling and participating in HCC progression [25]. However, the role of MAPKAPK5-AS1 in HCC is not fully clarified. This work confirmed that MAPKAPK5-AS1 expression was elevated in HCC tissues and cell lines, consistent with previous studies [25]. High expression of MAPKAPK5-AS1 was associated with larger tumor size, advanced stage and lymph node metastasis of the patients, and MAPKAPK5-AS1 could promote the proliferation, migration, invasion and EMT process of HCC cells.

To further clarify the mechanism by which MAPKAPK5-AS1 plays a role in HCC, through searching StarBase database, it was observed that MAPKAPK5-AS1 could probably modulate multiple miRNAs, and then miR-429 was selected for further research. A lot of studies confirm that miR-429 plays a tumor-suppressive role in many malignancies. For example, miR-429 can inhibit the proliferation, migration and invasion of nasopharyngeal carcinoma cells by inhibiting TLN1 expression [26]. Another study reports that, miR-429 expression is down-regulated in pancreatic cancer, and miR-429 exerts an effect on the migration, invasion and EMT of pancreatic cancer by regulating ZEB1 expression [27]. Similarly, as a tumor suppressor, miR-429 can directly target ZEB1 to inhibit the proliferation and induce the apoptosis of thyroid cancer cells [28]. In HCC, miR-429 can target CRKL to inhibit cancer cell migration and invasion by inhibiting Raf/MEK/ERK pathway [16]. Additionally, miR-429 represses HCC cell migration by targeting RAB23 [29]. Another study reports that, miR-429 expression is downregulated in HCC, and miR-429 inhibits HCC cell proliferation and migration by targeting TRAF6 and modulating NF-κB pathway [30]. Some previous studies have reported that MAPKAPK5-AS1 can function as a ceRNA to suppress miRNAs and reversely regulate the inhibitory effects of miRNA on target genes. Specifically, MAPKAPK5-AS1 promotes the proliferation and migration of thyroid cancer cells by targeting miR-519e-5p/YWHAH axis [24]. MAPKAPK5-AS1 regulates SNAI1 expression by sponging let-7f-1-3p and facilitates the progression of colorectal cancer [31]. In our study, luciferase activity reporter assay confirmed that MAPKAPK5-AS1 could directly bind with miR-429 and negatively modulate the expression of miR-429, and inhibition of miR-429 could partially counteract the effects of MAPKAPK5-AS1 knockdown on the proliferation, migration and invasion of HCC cells. Therefore, it is concluded that MAPKAPK5-AS1 might play a role in regulating the expressions of target genes in HCC as a molecular sponge of miR-429.

As a powerful EMT-related transcription factor, ZEB1 expression is found to be highly expressed in many malignancies, such as lung cancer, breast cancer and pancreatic cancer [32]. Importantly, ZEB1 is crucial regulator in the tumorigenesis and cancer progression of HCC. A previous research shows that the expression of ZEB1 in HCC tissues is significantly increased and its high expression is associated with the poor prognosis of HCC patients; ZEB1 silencing suppresses the the proliferation, migration and invasion of HCC cells by regulating vimentin expression [19]. Moreover, ZEB1 can promote the proliferation, migration and invasion of HCC cells via activating Wnt/β-catenin signaling pathway [33]. MiR-429 is probably involved in regulating the bone metastasis of breast cancer cells by targeting ZEB1 and CRKL [34]. Consistently, in this study, we found that ZEB1 3’UTR was a target of miR-429, and miR-429 could repress the expression of ZEB1 in HCC cells. It was also revealed that MAPKAPK5-AS1 could function as a molecular sponge of miR-429 to indirectly regulate ZEB1 expression. Our data partly explain the mechanism of ZEB1 dysregulation in HCC cells.

## Conclusion

In conclusion, this study reports that MAPKAPK5-AS1 expression is upregulated in HCC, and MAPKAPK5-AS1 acts as a tumor promoter to facilitate the proliferation, migration, invasion and EMT process of HCC cells by repressing miR-429 and upregulating ZEB1 expression*.* Our study represents a novel ceRNA network which is involved in HCC progression, and may provide useful clues for the diagnosis and treatment of HCC.

## Supplementary Information

**Additional file 1: Supplementary file 1.** The original photos of Western blot assays for Fig. 1 L&P, Fig. 2 G and Fig. 3 F (E-cadherin and N-cadherin).

**Additional file 2: Supplementary file 2.** The original photos of Western blot assays for Fig. 3 F (GAPDH), Fig. 4 K and Fig. 5 D&E.

## Data Availability

The data used to support the findings of this study are available from the corresponding author upon request.
